# Guidelines for Point-of-Care Fluorescence Imaging for Detection of Wound Bacterial Burden Based on Delphi Consensus

**DOI:** 10.3390/diagnostics11071219

**Published:** 2021-07-06

**Authors:** Alisha R. Oropallo, Charles Andersen, Raymond Abdo, Jenny Hurlow, Martha Kelso, Mark Melin, Thomas E. Serena

**Affiliations:** 1 Comprehensive Wound Healing Center and Hyperbarics, Department of Vascular Surgery, Zucker School of Medicine Hofstra/Northwell, Hempstead, NY 11549, USA; aoropallo@northwell.edu; 2 Wound Care Clinic, Madigan Army Medical Center Joint Base Lewis-McChord, Renton, WA 98431, USA; charles.a.andersen@civ.mail.mil; 3 St. Louis Foot & Ankle, LLC., St. Louis, MO 63109, USA; raymond.abdo@me.com; 4 Consultant Wound Care Specialized Nurse Practitioner, Memphis, TN 37501, USA; jenny.hurlow@gmail.com; 5 Wound Care Plus, LLC., Blue Springs, MO 64015, USA; martha.kelos@mywoundcareplus.com; 6 M Health Fairview Wound Healing Institute, South Campus, Edina, MN 55435, USA; melinmark7@gmail.com; 7 SerenaGroup Research Foundation, 125 Cambridge Park Dr., Cambridge, MA 02140, USA

**Keywords:** fluorescence imaging, bacteria, wound care, MolecuLight, Delphi method, consensus, guidelines

## Abstract

Excessive levels of bacteria impede wound healing and can lead to infectious complications. Unfortunately, clinical signs and symptoms of elevated bacterial burden are often unreliable. As a result, point--of--care fluorescence imaging, used to detect critical bacterial burden in wounds, is becoming widely recognized and adopted by clinicians across the globe as an accepted and added component of wound assessment protocol. A Delphi method was employed to establish consensus guidelines describing fluorescence imaging use. A multidisciplinary panel of 32 wound experts (56% MD, 22% podiatrist, 12.5% nurses/nurse practitioners) representing multiple sites of service (e.g., hospital outpatient, inpatient, private office, long-term care) completed two rounds of online questionnaires. The Delphi included key topics, including competencies required to perform imaging, clinical indications for imaging (e.g., signs/symptoms present, procedures warranting imaging), frequency of imaging, and a clinical workflow algorithm. Describing their clinical experiences of imaging impact, >80% reported changes in treatment plans, 96% reported that imaging-informed treatment plans led to improved wound healing, 78% reported reduced rates of amputations, and 83% reported reduced rates of microbiological sampling. The guidelines provided here will help to standardize use of fluorescence imaging among wound care providers and enhance the quality of patient care.

## 1. Introduction

Identifying and reducing or eliminating bioburden is fundamental to wound healing. However, the sensitivity of clinical examination is less than 15% and more than 80% of wounds with high bacterial loads remain undetected and incompletely treated [1,2]. These high levels of bacteria are known to impair wound healing rates [3,4,5,6]. As a result of a growing aging population, higher incidence of diabetes, and other chronic disease states, the prevalence of chronic wounds and their associated expenditure continues to rise. Chronic wounds are especially common among the elderly population, who have impaired immune function and frequently fail to mount clinical signs and symptoms of infection (CSS) [7]. 

In the US, Medicare spends USD 96.8 billion annually on chronic wound care [8]; the presence of an infection can further increase these costs by up to 70% [9]. Elevated loads of bacteria contribute to delayed wound healing, increase the risk of infection, lead to the failure of cellular tissue products (CTPs), and substantially increase the risk of sepsis and amputation [10,11,12,13,14,15]. A conservative estimate places the financial burden of elevated wound bacteria at more than USD 10 billion in annual Medicare spending. Improved methods of detecting bacterial burden are available and are essential to improving patient outcomes and reducing the significant economic burden of infected wounds. 

**Figure 1:**
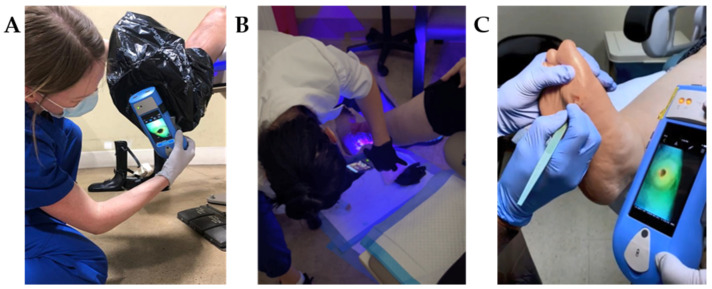
Fluorescence imaging procedure for wound bacterial presence, location and load performed across multiple sites of service. (**A**) When in a bright environment, a DarkDrape^®^ is attached and used to create adequate darkness. The drape is positioned around the anatomy and the fluorescence imaging device is positioned at an appropriate distance (8–12 cm) from the wound prior to focusing and capturing an image. (**B**) The DarkDrape^®^ is not required in a darkened environment. For large wounds wrapping around the anatomy, as shown here, multiple fields of view will be imaged and interpreted. (**C**) Fluorescence images can be immediately interpreted at the point-of-care to inform treatment. Multiple images may be acquired per visit to assess efficacy of treatments performed. This panel shows the image informing a physician’s debridement.

Point—of--care fluorescence imaging (MolecuLight *i:X*^TM^, MolecuLight Inc., Toronto, ON Canada) enables clinicians to noninvasively visualize the presence and location of bacteria at loads >10^4^ CFU/g. The procedure is performed at the patient’s bedside and does not require any patient contact. After preparing the wound (e.g., wiping any blood or debris [16]) and positioning the patient for imaging, a standard image in ambient room light is captured. The room lights are then turned off (or a DarkDrape^®^ is used) to create darkness needed to visualize fluorescence signals (See Figure 1). A light sensor on the device is used to confirm adequate darkness for imaging. The device is then positioned parallel to the wound. A range finder on the fluorescence imaging device ensures appropriate distance from the wound. The violet light on the device is switched on and clinician brings the wound into focus prior to capturing the fluorescence image. These steps may be repeated, as needed, during a single patient encounter. This imaging technology provides a direct, objective, and highly sensitive [1,17,18] method of identifying clinically significant bacterial levels in wounds. Wound care providers can utilize this information to immediately and appropriately manage bacterial burden to improve healing. When illuminated with violet light emitted from the imaging device, porphyrin-producing bacteria produce a red fluorescent signal while *Pseudomonas aeruginosa* uniquely fluoresces cyan [19,20]. These fluorescence signals are captured as point-of-care images or videos, which provide immediate information and documentation on bioburden. Clinical trials using quantitative tissue culture techniques as a reference standard demonstrate a high diagnostic accuracy of fluorescence imaging: >95% positive predictive value of red and cyan fluorescence in detecting bacterial loads known to delay healing [1,20,21], and a sensitivity that is 300–400% higher than the current standard of care assessment [1,2]. Clinical studies in “real-world” settings report improvements in wound healing, reduced amputation rates, and reduced need for antimicrobials and antibiotics (demonstrating improved antibiotic stewardship) when fluorescence imaging is incorporated into the care pathway [17,22,23]. Knowledge of bacteria location and load reveals the underappreciated impact of increased levels of bacteria in contributing to and driving wound chronicity [24].

The current study was developed for both clinicians and policy makers in a concerted effort to: (1) establish evidence-based universal guidelines describing the clinical indications for use of fluorescence imaging and (2) develop a prescribed clinical workflow demonstrating how to incorporate the fluorescence imaging procedure into standard of care to inform clinical decision making. 

## 2. Materials and Methods 

The Delphi technique is a well-established iterative, multi-round process used to collect knowledge and achieve expert consensus on a particular topic through at least two rounds of anonymous surveys [25]. This scientific method has been previously used by wound care experts to establish consensus on guidelines for NPWT with instillation [26], the use of dressings in chronic wound management [27] and development of clinical criteria for infection [28]. As part of the Delphi, experts on a particular topic are invited to put forward opinions in the first round and then indicate their extent of agreement or disagreement on aggregate data in subsequent rounds. This approach is repeated until an accord is established and summarized [29]. The advantages of this method include the ability to involve a large, international group of participants, maintain anonymity of respondents, and the capability of achieving consensus from experts on content informed by clinical evidence [30,31]. 

### 2.1. Expert Panel 

Thirty-nine wound care experts, chosen to reflect various professional groups across key sites of service where wound care is performed, were invited to participate in this independent, unsponsored Delphi consensus. All participants had firsthand experience with the technology. Two weeks prior to sending out the questionnaires, participants were recruited and were informed of the objectives of the Delphi process. Participant responses remained anonymous; individual responses were only known by the study moderator (TS). A subgroup of seven clinician experts formed the core expert panel involved in development of statements and a clinical algorithm (5 to 10 experts are considered adequate for content validation) [25]. Each core expert panel member had more than 15 years of experience providing wound care, and significant expertise using the fluorescence imaging procedure across multiple sites of service.

### 2.2. Delphi Survey 

For Round 1, 80 statements were prepared on clinical indications and frequency of performing the fluorescence imaging procedure. These statements were developed based on a review of the literature in combination with the clinical experience of the core expert panel. Participants were asked to select statements that, in their opinion, best reflected the clinical indications for use of fluorescence imaging. Participants were also asked to rate their agreement on a clinical workflow describing fluorescence imaging. A space was provided below each question to encourage participants to suggest additional criteria they felt were relevant to fluorescence imaging and its workflow, or to provide clarification on the items they selected. In Round 1, demographic information was also collected (licensed profession, years of experience, and duration of using the imaging device in their practice). In Round 2, responses from Round 1 that achieved consensus (>75%) were aggregated together focusing on various aspects of the imaging procedure (i.e., conditions or procedures warranting use of imaging, etc.) and participants were asked to review a revised clinical workflow diagram incorporating recommendations collected in Round 1. In Round 2, participants were also asked to report the real-world impact of imaging on their own patients’ wound outcomes by indicating their agreement using a 4-point Likert scale ranging from “strongly agree” to “strongly disagree”. Participants had the option of selecting “unable to comment” if they felt they lacked sufficient experience with the device to answer the question.

## 3. Results & Discussion

### 3.1. Delphi Participants

Invitations to voluntarily participate in the Delphi survey were sent by email to 39 wound care experts; a response was received from 32 experts, all of whom accepted the invitation. All experts participating were health care professionals providing wound care with >3 months experience using the fluorescence imaging device. Of the 32 healthcare professionals who participated in the Delphi, 84% were from the US, 78% were physicians and 63% had >15 years of experience in wound care (Table 1). Most participants worked in the hospital outpatient department (75%), inpatient department (62.5%), a private office (53.1%), and/or a long term care facility (18.8%) settings.

### 3.2. Delphi Results 

82% of invitees completed Round 1 (32/39) and 97% of clinicians who participated in Round 1 completed Round 2 (31/32), producing an average response rate of 89%. All initiated surveys had 100% completion for both rounds. In Round 1, 41 out of 80 statements reached consensus. Of the remaining 39 statements, 9 were near consensus (50–75%) and 30 items did not reach consensus (<50% agreement). In Round 2; statements that achieved or were close to consensus (>50%) were aggregated together to produce a total of 14 summary statements. 100% consensus was achieved for 4 of these summary statements. 

### 3.3. Recommendations 

#### 3.3.1. Competencies Required to Perform Fluorescence Imaging 

The competencies required to perform this procedure are unique and are not currently available through professional programs or courses. These competencies range from fundamental to advanced skills related to setting up the patient and device, how to use the device and how to interpret images. Competence in both fundamental and advanced skills are required to successfully integrate fluorescence-based information on bacterial load into treatment planning. This is especially true for image interpretation, which has a learning curve that improves over time. An online educational program with didactic and hands-on components is offered to new users prior to implementing the procedure in their clinical practice. This includes a free, interactive e-learning module on image interpretation through which clinicians can receive certification. 

Most experts agreed that these training components were necessary or critical to perform the imaging procedure, with consensus ranging between 93–100% after Round 1 for items listed in Table 2. This level of agreement suggests that the experts felt strongly that these competencies were necessary to effectively perform the imaging procedure. 

#### 3.3.2. Fluorescence Imaging Clinical Workflow 

**Figure 2:**
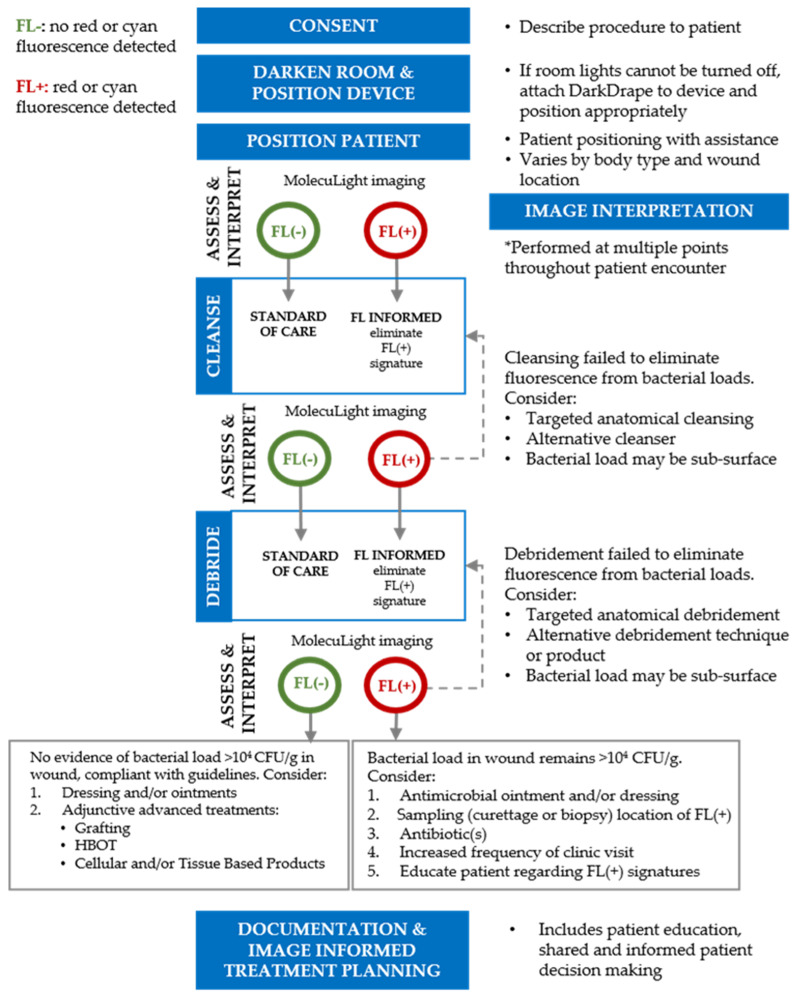
Clinical workflow for point-of-care fluorescence imaging. This clinical workflow describes the typical processes performed by clinicians when incorporating fluorescence imaging into their care pathway. This iterative approach provides clinicians with evidence of bacterial burden at the point-of-care and emphasizes use of clinical judgement to select actions that target removal of bacterial burden from the wound to facilitate wound healing. FL, fluorescence imaging; HBOT, hyperbaric oxygen therapy.

A clinical workflow (Figure 2) algorithm, based on prior publications demonstrating the benefit of similar fluorescence workflows in promoting healing [23,24], was developed. Iterative loops are a key component of the algorithm: images provide real-time feedback and assist in determining the effectiveness of wound care procedures such as cleansing and debridement. The clinical actions in the workflow and the multiple opportunities for assessment and interpretation enable clinicians to provide comprehensive care. 

Respondents agreed with the processes included in the clinical algorithm, with 81.3% of respondents indicating ‘agree’ or ‘strongly agree’ after Round 1. After Round 2, 93.5% of respondents indicated agreement. Many experts commented that the sequence of steps outlined in the workflow accurately represented their decision-making process and that fluorescence imaging “provides important information guiding clinical management decisions and testing”. Specifically, one respondent indicated “this workflow includes everything needed in the plan of care for a patient from assessment to education”. 

All participants agreed that the clinical algorithm described in Figure 2 may be used on a variety of wound types. There was 100% agreement among all 32 experts on use of this imaging procedure to assess diabetic foot ulcers. Similarly, high levels of agreement (80–97%) were obtained for other common chronic and acute wounds (Table 3). In addition to this list, there are less commonly occurring wounds (e.g., pilonidal sinus, pyoderma gangrenosum) for which a point-of-care diagnosis of bacterial presence and location, or absence, would have high clinical value [32,33]. 

#### 3.3.3. Clinical Indications for Fluorescence Imaging to Detect Bacterial Burden in Wounds 

The clinical indications for using fluorescence imaging were divided into 4 areas: (1) review patient medical history/prior treatment, (2) clinical assessment, (3) provision of interventions/treatments and (4) wound sampling. Experts agreed that the criteria for patient history and comorbidities warranted the use of fluorescence imaging. For example, it is well known that comorbid medical conditions, such as diabetes, can mask the presence of signs and symptoms of infection [11,34], and there is a strong association between delayed wound healing and presence of high bacterial burden [4,5,10,35]. The 100% consensus achieved for the patient history and comorbidity criteria suggests that clinicians strongly consider these criteria when deciding to perform fluorescence imaging.

Previous studies comparing fluorescence imaging to standard of care observed a high likelihood of elevated bacterial loads in wounds with few or no clinical signs or symptoms of infection [1,2]; therefore, the panel chose, with 96.7% agreement, that only one sign or symptom of infection needed to be present to indicate the need for imaging. However, it is not mandatory that any signs and symptoms be present, given that assessment of clinical signs and symptoms may miss the detection of >80% of wounds harboring high bacterial loads [1,3]. 

Point-of-care selection and monitoring of treatments to reduce or eliminate bacterial burden and promote wound healing has traditionally involved some degree of guesswork, albeit based on skilled judgement. Clinical assessment alone has a low sensitivity in detecting bacteria in chronic wounds [1,36]. In the absence of objective evidence of bacterial burden, clinicians must rely solely on proxy signs and symptoms to select a treatment. These treatments vary in cost and complexity and their selection may not demonstrate appropriate antibiotic stewardship. Many advanced therapies are contraindicated for use when high bacterial loads are present [12,37]. Cellular and/or Tissue-based Products (CTPs) for example, have an associated cost of up to USD 2000 to USD 10,000 per application, depending on size, and should not be used in the presence of red or cyan fluorescence in the wound. The use of fluorescence imaging concomitant with advanced therapies such as CTP application may increase their effectiveness and decrease inappropriate CTP application if inadequate wound bed preparation is evident [10,34]. Similarly, the ability to detect bacterial loads at the point-of-care with fluorescence imaging supports clinical judgement when selecting the appropriate intervention to manage bacterial burden in the wound [38,39,40]. In addition, clinicians can use the real time feedback provided by fluorescence imaging to assess the effectiveness of antiseptic therapy (i.e., dressings and topicals) over time. 

**Figure 3:**
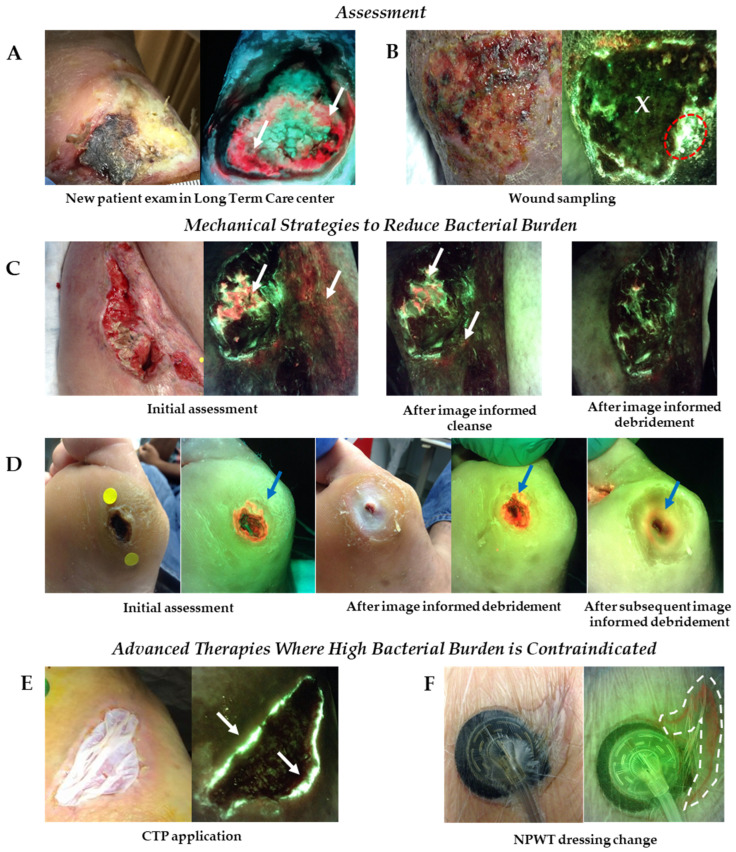
Use of fluorescence imaging across multiple sites of service. (**A**) Fluorescence images captured at initial assessment of a patient in long term care with a diabetic foot ulcer revealed significant bacterial burden around the wound (white arrows) that changed the plan of treatment put in place for this patient. (**B**) Images of a venous leg ulcer prompted clinician to collect wound biopsy from region of cyan fluorescence (denoted by red dotted circle), rather than the wound center (denoted by an ‘X’); sample was positive for *Pseudomonas aeruginosa.* (**C**) Patient with an incisional hip wound following surgery had red fluorescence (white arrows) from bacteria in and around wound. The wound was washed vigorously (HOCL cleanser) and re-imaged. Persistence of red fluorescence prompted additional cleansing and debridement in regions of red fluorescence. Red fluorescence diminished after image-informed mechanical strategies. (**D**) Red fluorescence from bacteria was detected in a diabetic foot ulcer, highlighted by blue arrow, prompting debridement. Initial debridement revealed a larger amount of bright red fluorescence in the callus tissue surrounding the wound. Additional debridement was performed to remove bacteria laden tissue. (**E**) A diabetic foot ulcer that had previously received multiple CTP applications underwent fluorescence imaging prior to application of another CTP. Images revealed the presence of cyan fluorescence (white arrows) indicative of *Pseudomonas aeruginosa* around the wound edge; CTP application was withheld until cyan signal was eliminated. (**F**) NPWT was applied to an appendectomy abscess. Fluorescence images were captured during a dressing change and indicated presence of red fluorescence around the wound (denoted by dashed white dotted line). Imaging informed mechanical bacterial removal prior to placement of a new dressing. CTP, cellular tissue product; NPWT, negative pressure wound therapy.

Included in this grouping of procedures in Table 4 was the use of imaging for initial assessment of wounds from patients in long term care (LTC) (Figure 3A). Experts working in LTC indicated that immunocompromised elderly patients may not exhibit signs and symptoms of infection; the first evidence of wound infection in these patients is often sepsis [41,42]. Therefore, the use of fluorescence imaging at the initial patient encounter may enable wound care providers in this setting to identify at risk patients earlier and reduce risk of invasive infection.

There was a high degree of consensus among experts regarding the procedures and treatments listed in Table 4 that may warrant fluorescence imaging. Of note, for many of these procedures and treatments, experts agreed that fluorescence imaging may be performed before, during and/or after the intervention. Figure 3C,D provide clinical examples of fluorescence imaging used prior to, concurrent with or following mechanical strategies to reduce bacterial burden. Figure 3E,F show use of imaging to provide immediate, actionable information on bacterial burden prior to or following placement of advanced therapies including skin substitutes or grafts and NPWT where high bacterial burden is contraindicated.

The capture of standard images prior to fluorescence images enables clinicians to identify and use anatomical markers or unique characteristics of the wound identified on the standard image (e.g., proximity to medial malleolus or shape of the wound edge) as landmarks when interpreting fluorescence images. For example, in Figure 3B, after capturing the fluorescence image, the clinician then reviewed the standard image and compared to the fluorescence images. The irregular shape of the wound edge on the bottom right corner of the wound observed in the standard image was consistent with the pattern of cyan fluorescence (indicative of *Pseudomonas aeruginosa*) in the fluorescence image. With this information, the clinician was able to narrow down the region of the wound that was most optimal to sample for microbiological analysis. Of note, the range finder function on the device also enables the clinician to image the wound from approximately the same distance as in prior visits. 

In instances where red or cyan fluorescence persist after debridement (or other therapies), a wound sample for microbiology may be warranted. More than 87% of experts agreed that fluorescence imaging may be used to inform the location of sample collected or referral for microbiological analysis. This is consistent with prior studies demonstrating decreased false negative sampling results with fluorescence-informed sampling location [43]. In addition, studies have consistently shown that sampling in regions positive for red or cyan fluorescence will detect elevated levels of bacteria >95% of the time [1,17,18,20,21]. Sampling for microbiology provides information on the species of bacteria present and antibiotic sensitivities, enabling appropriate selection of an antibiotic when warranted, contributing to disciplined antibiotic stewardship [44,45]. An example of sampling from a region of cyan fluorescence is depicted in Figure 3B. 

#### 3.3.4. Recommended Frequency of Fluorescence Imaging 

One of the most common questions that arise when clinicians first adopt this diagnostic imaging technology is “how often should I image my patients?” Unlike other imaging procedures (e.g., X-rays) that pose a risk to the patient with prolonged exposure, fluorescence imaging uses a safe, visible violet light to detect bacteria; as such, imaging can be performed repeatedly without any detriment to patients. Typically, clinicians capture a fluorescence image at the initial patient encounter (baseline) to determine the status of the wound and identify additional tests or treatments that may be needed. At subsequent patient encounters, >93.5% of experts agreed that fluorescence imaging should be performed no more than weekly, unless otherwise clinically indicated (Table 5). This frequency was determined based on the understanding that many prescribed interventions aimed at targeting bacterial burden and promoting wound healing (e.g., antimicrobial dressings) take days or weeks to observe an effect. This frequency also matched the typical frequency at which patients visit wound care centers for follow up visits. The time intervals may decrease (i.e., more than once a week) or increase (i.e., evaluations every 2–4 weeks), depending on the progression of the clinical status. The core panel emphasized the importance of medical necessity and use of clinical judgement to avoid unnecessary procedures or treatments while ensuring that patients receive the optimal care needed to heal their wounds. 

### 3.4. Reported Impact of Fluorescence Imaging on Treatment Planning & Wound Outcomes 

**Figure 4:**
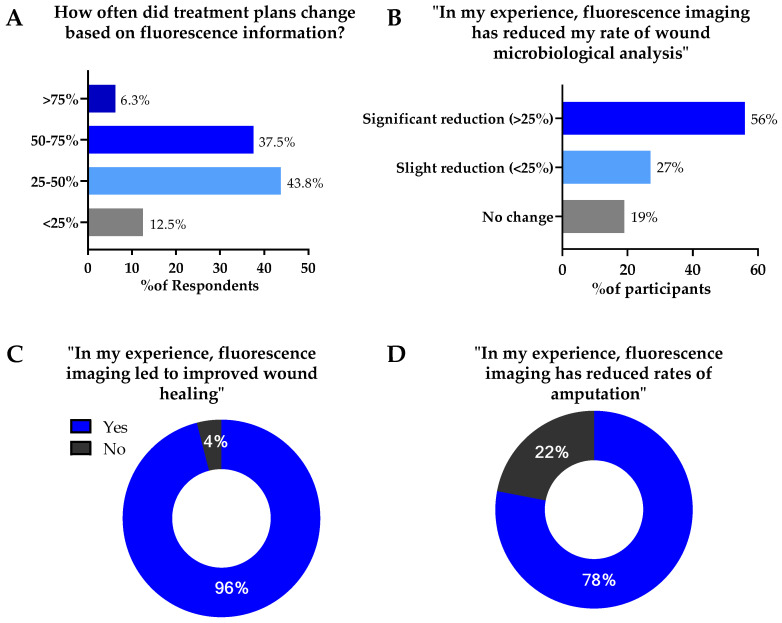
Impact of fluorescence imaging of bacteria on treatment protocols and wound outcomes. A total of 31 participants provided responses. (**A**) Percent of Delphi participants reporting changes in treatment plans based on fluorescence imaging. (**B**) Percent of participants reporting reduction in wound sampling for microbiological analysis because of point—of—care fluorescence imaging information. (**C**) Percent of participants indicating improvements in wound healing due to use of fluorescence imaging. (**D**) Percent of participants indicating reduced rates of amputation due to use of fluorescence imaging.

This consensus provided a unique opportunity to receive feedback from a group of wound care experts with significant experience implementing fluorescence imaging into their clinical care pathway. To better appreciate the potential impact of the objective information provided by fluorescence imaging, several questions were included in the Delphi, assessing the impact of fluorescence imaging on treatment planning and wound outcomes. Most experts (87.6%) indicated that the objective information provided by fluorescence imaging changed their treatment plans in a significant number of cases (Figure 4A). These findings are consistent with results from a clinical study of 350 patients in which changes to treatment plans following capture and review of fluorescence images were observed in 70% of patients [1]. A similar response was observed when experts were asked to report the impact of fluorescence imaging on rates of wound microbiological sampling. 83% of experts reported that fluorescence imaging reduced their rate of microbiological sampling, of which 56% reported a significant reduction (>25%) (Figure 4B). Panelists agreed that there will be times when sampling information is critical, and that fluorescence imaging cannot replace sampling in terms of confirming antibiotic susceptibilities and speciation. The reduced rate of sampling reported by experts indicates that many felt confident in using fluorescence imaging information to monitor changes in bacterial burden over time. 

Included in the Delphi were questions on the reported impact of fluorescence imaging on patients’ wound outcomes. 96% of experts reported that, in their experience, imaging led to improved wound healing (Figure 4C). When asked about rates of amputation, 78% of experts reported reduced rates of amputation with use of fluorescence imaging (Figure 4D). These reports are in line with recent studies evaluating the impact of fluorescence imaging on wound outcomes. A 2-year retrospective analysis of 229 foot ulcers reported a 23% increase in wound healing rates with incorporation of fluorescence imaging. These improved wound healing rates were attributed to earlier bacterial detection and more thorough debridement performed at the point-of-care, using objective fluorescence information [23]. Multiple recent randomized controlled trials (RCTs) have also demonstrated substantial improvements (>200%) in 12-week wound healing rates when fluorescence imaging is incorporated into standard of care [46,47]. In these RCTs, improvements in wound healing rates occurred without increases in antibiotic prescribing and without addition of CTPs, suggesting a substantial decrease in cost of care. These findings suggest that by following a workflow as described in Figure 2, patients may receive more optimal treatment from their wound care providers, contributing to improved outcomes as described in Figure 4. 

## 4. Conclusions

Consensus on guidelines for the use of fluorescence imaging was obtained through a two-round Delphi process. The final product includes a series of guidelines (Table 2, Table 3 and Table 4) and a clinical workflow (Figure 2) that provides a framework for multiple stakeholders including clinicians, medical, surgical, and wound care societies, policy makers and regulatory bodies. Evidence indicates that use of consensus guidelines improves the quality of healthcare provided by supporting evidence-based, best-practice care. Furthermore, development of a clinical workflow algorithm provides a stepwise sequence that improves the consistency and coordination of care across the wound care continuum [25]. 

The topics included in the Delphi consensus address many common concerns that new users encounter when adopting this technology. For example, information on clinical indications and frequency of imaging can aid clinicians in understanding the medical necessity needed to perform this procedure. Statement development was based on peer-reviewed publications in addition to the clinical experience of a diverse panel of wound care experts familiar with the fluorescence imaging procedure. There was a high level of agreement (>85%) among wound care experts familiar with the fluorescence imaging procedure (well above the typical threshold for most consensus statements), indicating a high level of support for the adoption of these clinical indications. A summary of guidelines based on the result of this Delphi consensus is listed in Table 6.

The high level of agreement among experts demonstrates the utility and value of this diagnostic technology in the management of chronic wounds. Use of this imaging procedure enables healthcare providers to rapidly detect elevated levels of bacteria and develop an effective treatment plan while at the same time avoiding the use of advanced therapies when they are contradicted. In addition to improving patient outcomes, this technology has high potential to reduce the overuse of antimicrobials and reduce healthcare costs for both patients and health systems. 

## Figures and Tables

**Table 1:** Participant Demographics.

Country		Licensed Profession	
USA	84.0%	Medical doctor	56.3%
Canada	6.3%	Podiatrist	21.9%
United Kingdom	9.4%	Nurse Practitioner	9.4%
		Physical Therapist	3.1%
**Sites of Service**		Nurse	9.4%
Hospital Outpatient	75.0%		
Hospital Inpatient	62.5%	**Years of Experience in wound care**	
Private Office	53.1%	>20	40.6%
Telehealth	34.4%	15 to 20	21.9%
Long Term Care Facility	18.8%	10 to 15	25.0%
Long Term Acute Care Hospital	15.6%	5 to 10	6.3%
Home Health	9.4%	0 to 5	6.3%
Other (i.e., Mobile Unit, Urgent Care)	37.5%		

**Table 2:** Competencies mandatory to perform fluorescence imaging of bacterial burden.

Fundamental Competencies		Advanced Competencies
▪ Setting up device (imaging in focus, download software, exporting images) ▪ Capturing images (focusing) ▪ Understanding range finder and light indicators ▪ How to position and distance the patient and device appropriately ▪ Adequate lighting conditions ^1^	▪ How to interpret images with red fluorescence ▪ How to interpret images with cyan fluorescence	▪ Using image interpretation to plan treatment ▪ Aligning image interpretation to location of elevated levels of bacteria

^1^ Darkness is required and achieved by turning off the lights or using a DarkDrape.

**Table 3:** Wound types where fluorescence imaging of bacterial burden may be indicated.

Fluorescence Imaging May Be Performed on (but Not Limited to) Any of the Following Wound Types:
Diabetic foot ulcer Venous leg Ulcer Pressure Ulcer Surgical Site infection Post-operative wound Traumatic wound

**Table 4:** Recommended use of fluorescence imaging for detection of bacterial burden.

New Patient Exam
**Prior history or comorbidities**
History of delayed wound healing (>4 weeks) History of wound infection Failure of prior treatment Positive for clinical signs and symptoms present at visit Co-morbid medical conditions that mask signs and symptoms (i.e., diabetes, autoimmune disorders, elderly) Multiple co-morbid medical conditions that elevate risk of life/limb loss
**At least one of the following signs or symptoms**:
Delayed wound healing Wound breakdown and enlargement Increased malodor Local warmth New or increase pain Erythema Purulent discharge Extending induration Lymphangitis Crepitus Bleeding, friable granulation
**Procedures or treatment**
Follow up on positive fluorescence image Prior to, concurrent with or after debridement Suspected or confirmed biofilm Prescription of antimicrobials (including antibiotics) Follow up on application of antimicrobial Prior to or follow up on CTP/graft application Prior to, concurrent with or after NPWT Baseline assessment of new wound/patient in LTC/nursing facility
**Wound sampling**
Follow up on positive microbiology Inform need for and location of microbiological sample Inform referral for microbiological analysis

**Table 5:** Recommended frequency of fluorescence imaging.

Recommended Frequency of Fluorescence Imaging.
Fluorescence imaging may be performed on wounds at baseline, during the first 4 weeks and/or if an increase in wound size is observed
Fluorescence imaging may be performed weekly on patients that meet criteria for imaging (e.g., clinical signs and symptoms)
If a wound is positive for fluorescence, fluorescence imaging is performed no more than on a weekly basis; unless otherwise indicated by development or change in symptoms

**Table 6:** Summary of guidelines based on Delphi consensus.

Summary of Guidelines for Fluorescence Imaging
Training is needed to effectively perform fluorescence imaging. E.g., how to position the patient and device, how to interpret images The clinical workflow for fluorescence imaging compliments and is in addition to clinical wound assessment and treatment. Clinical judgement should be used to apply fluorescence information to treatment decision making. Medical history, comorbidities and signs of infection may inform the medical necessity for fluorescence imaging. E.g., history of delayed wound healing, presence of diabetes, detection of pain or malodor Fluorescence imaging may be performed prior to, concurrent with, or following many common procedures and therapies in wound care. E.g., Debridement, CTP application, prescription of antimicrobials, wound sampling Fluorescence imaging should be performed no more than weekly, unless otherwise indicated by medical necessity.
